# Gender differences in research fields of bioeconomy and rural development-based on sustainable systems in Latin America and Africa regions

**DOI:** 10.1371/journal.pone.0308713

**Published:** 2024-08-22

**Authors:** M. Lourdes Ordoñez Olivo, Rachael Adeleye Oluwakemi, Zoltán Lakner, Tibor Farkas

**Affiliations:** 1 Faculty of Economics and Social Sciences, Hungarian University of Agricultural and Life Science, Gödöllo, Hungary; 2 Institute of Rural Development and Sustainable Economy, Hungarian University of Agricultural and Life Science, Gödöllo, Hungary; Cranfield University, UNITED KINGDOM OF GREAT BRITAIN AND NORTHERN IRELAND

## Abstract

Using bibliometric analysis of large-scale publication data is a simple approach to exploring gender-related trends, especially gender equality in academic publishing. The aim of this study is to investigate gender trends in the fields of bio-economy and rural development sciences in two under develop regions as Latin America and Africa. This study examines gender differences in these fields in order to: (1) recognize the contribution of female researchers in bioeconomy and rural development, (2) explore the relational structure of gender aspects in academic publications, (3) identify trends in female authorship in these scientific research fields over time, and finally (4) identify gender potentials for women to become more visible in these fields of study. To achieve these objectives, we used bibliometric tools to analyses 1891 publication records in bioeconomy and rural development. After cleaning the database of full names of authors of academic publications relevant to the field studies, we performed a series of statistical analyses in R and SPSS software, such as Lotkas distribution, network analysis, co-authorship analysis and spatial distribution of authors in the study. The results show that the number of male authors is almost three times higher than the number of female authors, suggesting that women are under-represented in the fields studied. Men occupy the most important position of authorship in scientific articles; publications with corresponding male authors were found in 1389 out of 1891 publications related to the bio-economy and rural development. In terms of geographical regions, publications with female authors were more prevalent in European and North American areas, with a small exception in some developing countries such as Argentina and South Africa. In terms of research networks, from the total number of authors evaluated, only 23% are female authors on the map of research influence. This indicates that there is a significant gap to be filled in the promotion of scholarly impact through the sharing of knowledge and expertise among authors.

## Introduction

### Women’s share in academic publication

Gender equality has been in the limelight of global concern, and this is evidenced in the Sustainable Development Goals (SDG 5), with the main aim of achieving gender equality and empowering all women and girls by 2030 [1]. Despite all these set goals, gender inequality remains inherent in almost all social and economic aspects and, by extension, seen even in the scientific world among researchers where males and females do research; more are underrepresented in prominent scientific leadership positions [2].

Researchers are professionals involved in the conception or creation of new knowledge. They conduct research and expand or develop concepts, theories, models, methods, instrumentation or operational systems in the framework of research and development projects [3]. Discrepancies have been observed over the years of the place and role of women in science as against their male counterparts [4], and later became a standard knowing that “Women researchers and scientists have been long underpromoted, underrepresented and underpaid in their fields” [5]. UIS data in 2019, shows that less than 30% of the world’s researchers are women and the extent to which these women work in public, private, or academic sectors and their fields of research [6]. Overall, recent studies confirm the existence of gender inequality in science, which may be related to socio-economic characteristics, demographic location and other factors that contribute to the emergence of gender gaps [7, 8].

### Women share in bioeconomy and rural development research

There has been a growing interest in the bioeconomy as one of the sustainable global development solutions. This has been reflected in implementing several national and international strategies and policies in more than 50 countries and international organizations [9]. According to [10] promoting women in research and leadership positions contributes to building a sustainable bioeconomy at the European level. The statement is also reinforced by the negative influence that gender inequality has on the development of the bioeconomy [11].

The Gender Impact Platform [12], points out that gender researchers can generate data needed to restored embody women’s realities and build evidence to inform policy that can reduce social and economic inequality, which is also part of the main goals of bioeconomy and rural development. The researchers that collect, analyze, and share these results at the policy level can support women’s empowerment and their links to sustainable development. For the Europe Commission in the last Statement on International Women’s Day in 2018, "gender equality in all the aspects of the life of a woman is not just about fairness and justice, it is also a necessity to achieve sustainable peace, security, development, economic prosperity and growth around the world"[13].

### Bibliometric approach

The concept of bibliometric analysis has long been defined by various authors, such as [14], who describes it as “the application of statistical and mathematical methods to books and other media”. For others, it is a scientific field that uses quantitative means to assess scientific productivity [15], and identify core research or authors and their relationships, covering all publications in a given field [16]. Today, bibliometric methodology comprises components from mathematics, social sciences, natural sciences, engineering and even life sciences [17]provide a systematic description of the structure of research and focus on the quantitative aspects of scientific research output [18].

Bibliometric analysis has gained immense popularity in recent years [17, 19], and its popularity can be attributed to; the advancement, availability, and accessibility of bibliometric software such as R packages, Leximancer, Gephi, VOS viewer, and the use of the scientific databases such as Scopus and Web of Science [20]. There has also been an enhancement in the knowledge of gender inequality in the research and academic field lately due to the advancement in the use of different analytical methods like bibliometrics. Large numbers of bibliometric analyses have been done in recent years to examine sex disparities among literary production in different fields of studies looking at the contribution of women in these areas such as sport [21], economy [22], medicine [23], and social sciences [8, 24–26].

Bibliometric analysis is done based on specific indicators to each field of science, thus applying mathematical and statistical methods to describe the different aspects of scientific communication. The main elements of bibliometric analysis have been defined as database compilation, consistency and accuracy of the data, data fields, search options, and analysis and use of metrics [27]. The application of contemporary bibliometric principles covers three areas: methodology research, scientific disciplines, and science policy [17]. In this study, we focus on the applications of bibliometric methods to determine the gender balance in bioeconomy and rural development research fields which can be categorized under the sub-area of scientific disciplines. Previous studies have reviewed and carried out research on different aspects of bioeconomy and rural development as it affects climate change [28–30], environment [31], among others. However, in the field of bioeconomy and rural development, academic publications have not focused on gender differences among researchers. This is an essential aspect of gender balance and equality, with a significant focus on female contributions and how it can be more inclusive.

Sequel to the above, these researchers sought to answer the following questions:

1. What is the contribution of women researchers to the bioeconomy and rural development fields?
2. What are the women authorship trends in bioeconomy and rural development research over time?
3. What is the relational structure of gender aspects, research fields, and methodological features in both regions analyzed?
4. What is the extent of recognition and impact of women in the related literature?
5. How can women in science become more visible in these fields of study?

In summary, by conducting a network bibliometric analysis of the articles published so far, this study aims to provide a comprehensive intellectual structure of the current research landscape and discuss the most popular research topics in bioeconomy and rural development and their evolution over time based on sustainable systems in Latin America and Africa regions. The results of this study will provide not only a generic intellectual overview of women’s participation in these fields, but also an overview of how these trends is growing and the likely prospects for women scientists. In addition, through this study, we could highlight phenomenological ways in which women can participate in future research and ultimately achieve gender equality.

## Materials and methods

Given that this study focuses on the role of women in the bioeconomy and rural development based on sustainable systems, it is crucial to consider the overall context. To analyze trends in the proportion of these publications and the absolute number of articles, we took the bibliographic dataset from Web of Science (WoS), one of the world’s most recognized academic databases [32, 33].

The construction of the relevant bibliometric corpus was an encounter in several aspects. The first challenge was to identify research results in the field of bioeconomy and rural development, as both span multiple scientific fields, and a significant proportion of the research is interdisciplinary. The second challenge was identifying the authors gender difference from bibliometric information, considering that the raw dataset obtained by the different software does not make any distinction

### Data collection

The first steps for the data collection were an internal discussion with the authors to determine which terms related to the main two fields were important to consider and the scope of the geographical area the research will cover. We consider that the applied boolean operators in the database group the criteria previously discussed by the authors.

The primary documents of the data collection "core dataset" were obtained from the WoS database, which is widely used in meta-analysis studies [32, 34]. The following query was applied in the research: TS = ((("bioeconom*") OR ("Rural development*") OR ("Green economy*") OR ("CIRCULAR ECONOMY*") OR ("Regional development*") OR ("Agricultural development*")) AND (("africa*") OR ("Latin America*"))).

The data set included 2585 items (articles) which were refined by different criteria; firstly, the type of documents in this case, we did not consider enriched cited reference, associated data, meeting data, or letters; secondly, the language of the documents considered the ones written in English, Spanish or Portuguese.

Apart from the mentioned criteria, from the original data set, we extracted just the ones where the authors’ full name was indicated since we have not been able to uncover any pattern or indication of the authors’ given names. Therefore, we considered these articles as an unbiased estimation of the original dataset. The last presumption is essential since the main objective of the research is to determine the gender distribution in the data set.

**Fig 1:**
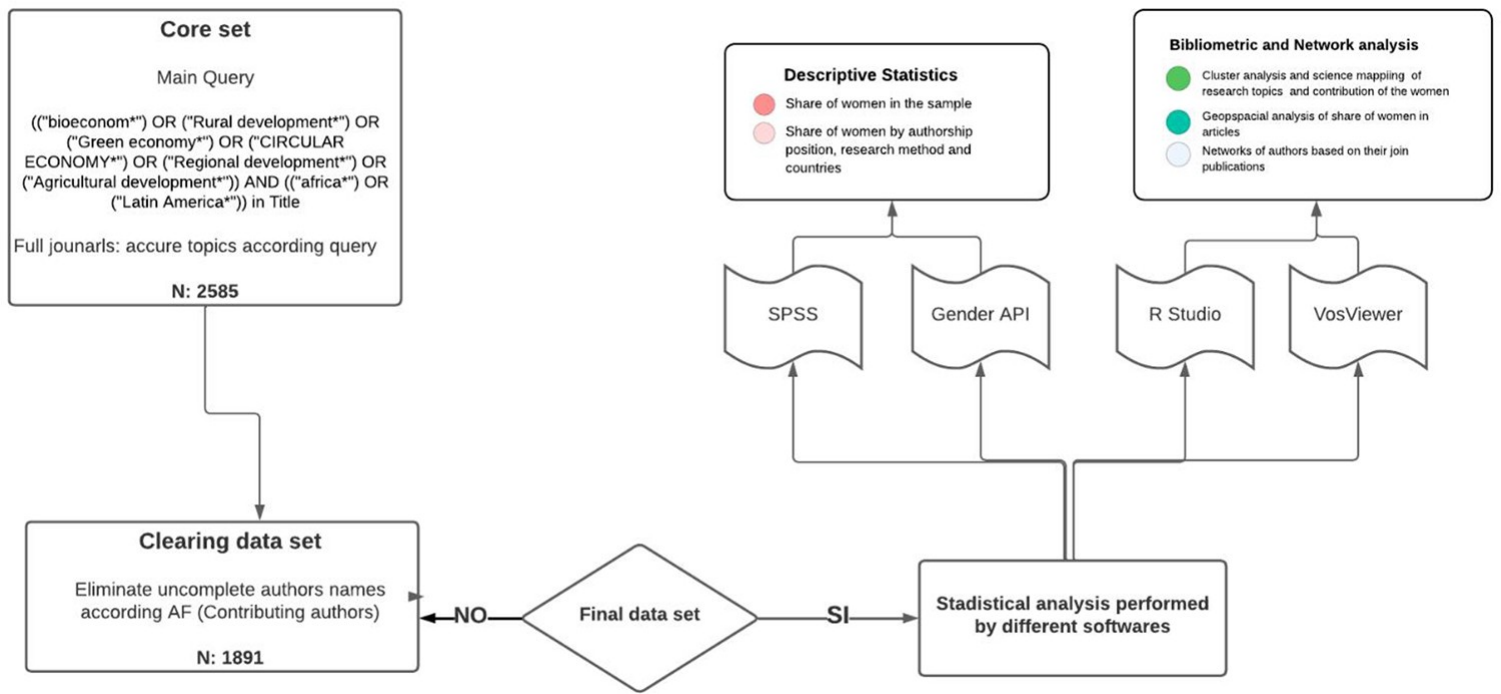
Research workflow. **Figure** shows the different steps in the research process, from obtaining the core set to the statistical presentation of the results. The figure created using lucid chart software.

After the mentioned criteria were applied, the final corpus contains 1891 publications, with dates of publications ranging between the beginnings of 1976 and September 2022. Fig 1, illustrates the identification workflow of relevant research papers.

### Gender identification

As a first step toward gender identification, the incomplete name of authors was subtracted based on the field tag "AF" [35] or called full author names retrieved from the bibliographic data downloaded from the WoS databases, which contains a dedicated field for representing the complete name of contributing authors. We used this parameter to eliminate authors where only the initials of the first name were provided.

For the second step for identifying the gender of the authors, we used the website Gender Api [36], which is considered one of the biggest platforms on the internet which determine gender by the full name or email address. They implemented a multi-layer technology to provide the most accurate results and performed several normalizations on the name to fix typos to cover all spelling variants. In general, their database contains 6,084,389 validated names from 189 different countries.

The platform Gender API provides an accuracy value between 0 and 100% in the query result. The final dataset contained 4,820 author names, from which we could identify 4,732 full author names. Of these, 4,091 (84%) authors’ gender was assigned with an average accuracy value of 98.4%. In the case of the unknown values, where the first or middle name was abbreviated, we completed the names and genders by secondary research on different scientific platforms as LinkedIn and google scholar. To corroborate the gender category, we verified in the sample of the data set the top 50 authors according to their profiles.

### Data analysis

The data analysis was made in two phases. The first and main one is based on the bibliometric analysis of documents registered on the WoS database set of the research [37, 38]. Based on the different publications, we classified the topics based on the country, research fields, funding information, and the determination of the role of women in different papers. Data analysis included geospatial analysis which determine the contribution of women by geographical areas, cluster analysis [39] to uncover emerging topics as well as the number and authorship position of women in them; and finally the co-occurrence network based on Spinglass Clustering Algorithm [40] analysis to create the authorship figures to examine the presence and role of women in the network.

Furthermore, in the second phase of the bibliometric analysis, we analyzed the statistical characteristics of the database obtained [41], namely: the distribution of authors by number of articles, the comparative position of authorship by gender, the thematic structure of science in the fields of bioeconomy and rural development and the contribution of women in these fields, the degree of centrality and density among the networks (clusters) and the network of co-authorship analysis. In reference to the latter two analyses, for the first case centrality refers to the metric that measures the correlation with other networks, themes and topics among the fields of study [41, 42]. While the dimension of intra-network represents the interactions with a robust internal relationship to a topic being referred to as density [43]. For the second analysis each node represents an author with a certain number of publications, the node size indicates the number of published articles, and the link thickness represents the intensity of cooperation[39, 44].

To make the data analysis as complete as possible, we used different statistical software to help us process the data. For the bibliometric analysis [45] we used R programming language as a basis and R-tool bibliometrix to interpreted the results[46–48]; for the cluster analysis, as the development of the research field over time, we processed the data in Vos Viewer [49–51] and the general descriptive statistics of the primary data set was development by SPSS [52, 53].

The present study does not necessarily require ethical approval, as the data analyzed correspond to public information contained in published scientific articles in which the authors are identified. The articles analyzed in the database were obtained from the Web of Science library and, once the authors of the scientific articles were identified, mathematical and statistical analysis techniques were used to differentiate the sex of the authors, which is explained in the methodological section of the manuscript. The main search terms were included in the manuscript so that reviewers or interested parties could obtain the database criteria used and authors information.

### Limitations

During the keyword search process, after following the guidelines in Fig 1, we noted that we had achieved the type of keyword combinations that were robust and firm enough to achieve the results to be displayed. The previous statement indicates that the subtraction or addition of a relatively less relevant keyword could not considerably influence the number of results. However, we are aware that our research does not necessarily cover all relevant articles on these topics.

One of the starting hypotheses of the research conducted is that we assume that the participation of women in academic articles is proportional to their role in research. However, we understand that the reality of this assertion will need to be validated by more erudite qualitative methods in future studies.

Finally, we should point out that we have identified the gender of the authors from their names. However, we know that this accuracy may be disputed since, for the study, it was the only variable that defined the authors’ identity. Despite these present research shortcomings, we expect that our results shed light on the existing problem. This will motivate further research studies for a better understanding of the participation and role of women in the bioeconomy and rural development studies.

## Results

**Fig 2:**
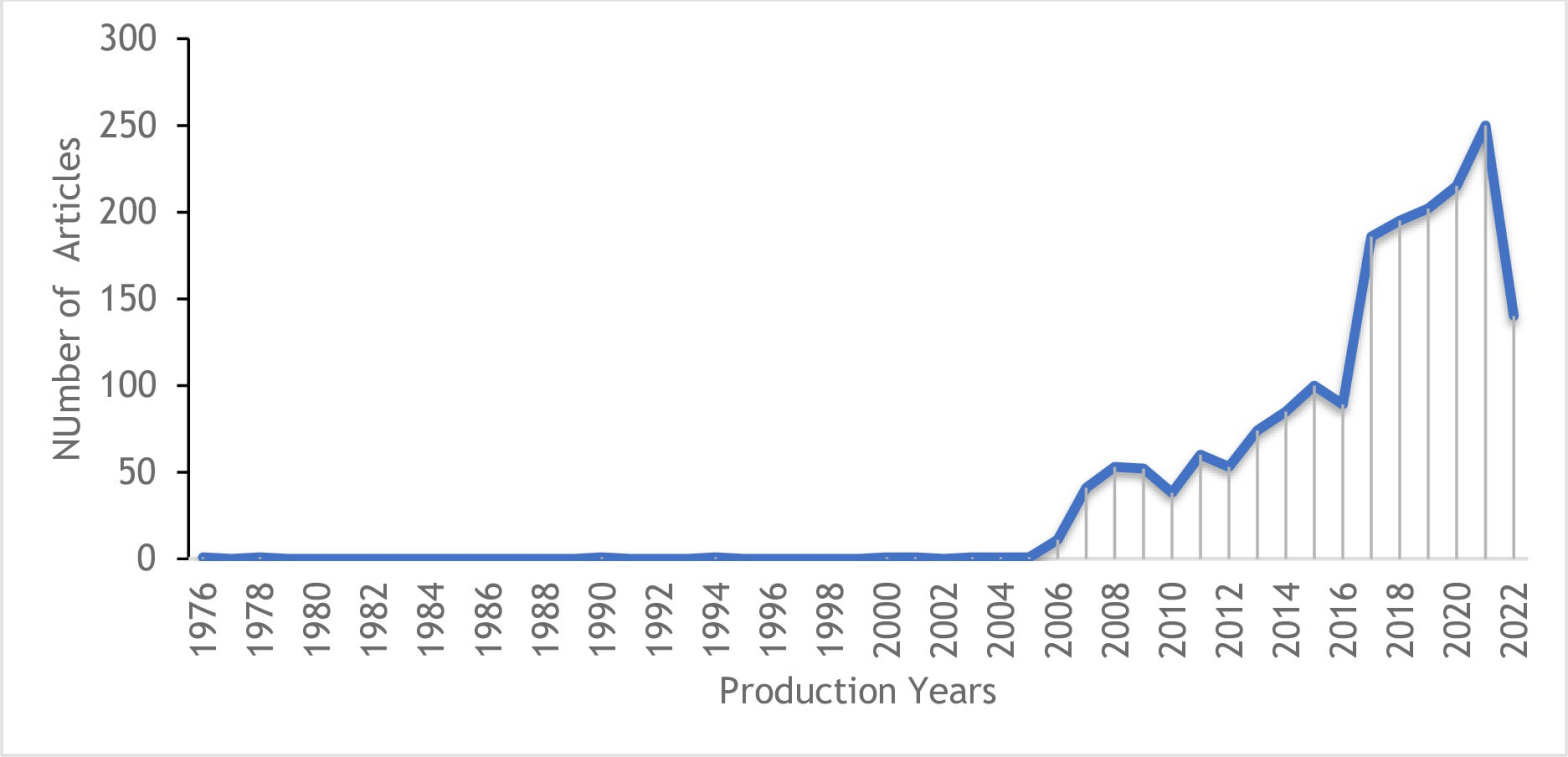
Evolution of the research output between 1976 and 2022 in the bio-economy and rural development in the countries of Africa and Latin America. The double axes represent the historical scientific production of articles generated during 46 years in the fields analyzed in this study.

Fig 2 presents the exponential trend in the increase of publications in the research fields studied between 1976 and 2022. During the first two decades, the number of publications on these topics is insignificant compared to the following years; from 2007 onwards, the publications have increased, reaching the highest values in the last years.

### Features of the database

The dataset consists of 1,891 documents. Approximately two-thirds of the publications have been researching articles (1,512); the remaining proportion is distributed among reviews (n = 161), proceedings papers (n = 81), and interprofessional discussions (n = 38), among others, which we took into consideration due to the complexity of the fields. In total, 4,835 authors were included in the fundamental corpus. The mean number of citations is 17.23 times, and 396 outputs (21% of the total number of publications) are single-authored, on average, 3.41 co-authors were found in the document. All these parameters indicate the complexity of the fields studied.

Based on the frequency of publications in the countries of the corresponding authors (intra-CCP and inter-CCP), more than 33% of the articles were produced in the United States and some European countries (United Kingdom, Germany, and Netherlands). It is also noteworthy that South African authors published almost 15% of the data set. The participation of Latin American authors is lower (8% of the total number of publications) compared to Europeans or North Americans; the most prominent countries in the region have been Colombia, Brazil, Mexico, Argentina, Chile, and Ecuador. South Africa has the highest participation in the African region, followed by Kenya, Ghana, Ethiopia, and Nigeria.

**Fig 3:**
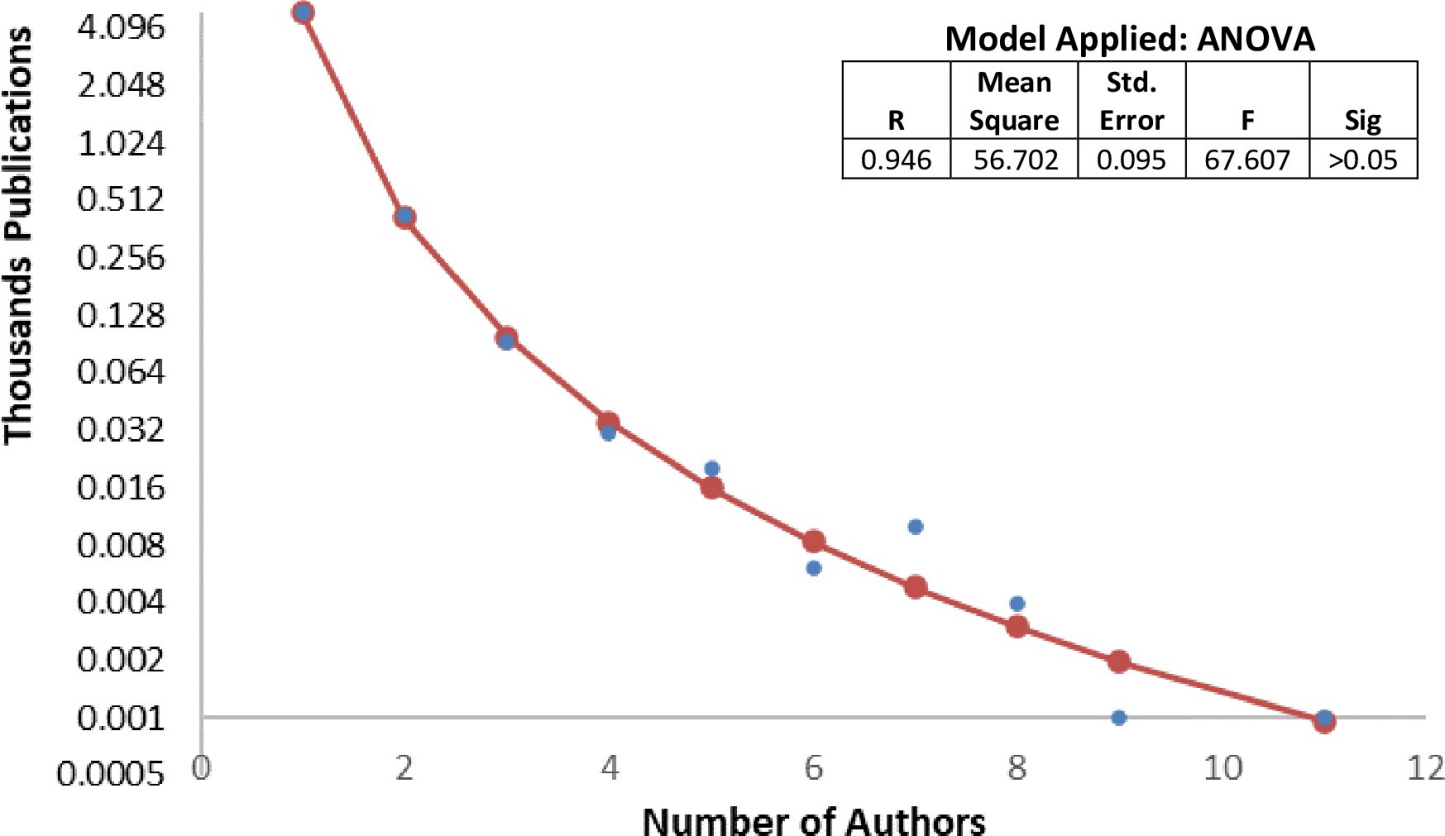
Distribution of all contributors by number of papers. On the basis of a two-axis analysis of the number of authors and the number of publications in the data set, we have shown the distribution of authors according to the number of publications. As a supplement to the figure, we used the simple ANOVA test for the determination of statistically significant differences between the means of the two groups of variables.

Fig 3 shows the distribution of authors according to the number of publications, which is obtained from the data generated by the Lotka’s model [53]. Most authors (89%) appear only once in the corpus; up to 42 authors produced more than five articles. Within the statistical analysis (ANOVA) applied of the above values, the r-squared value was 0.946 and the significance value was less than 0.05.

### Contribution of the women in the research fields

Regarding the contribution of women in the research fields, the number of male authors in the data set was almost three times greater than the number of female authors, with 1,496 identified as women and 3,324 identified as males (S1 Table).

**Fig 4:**
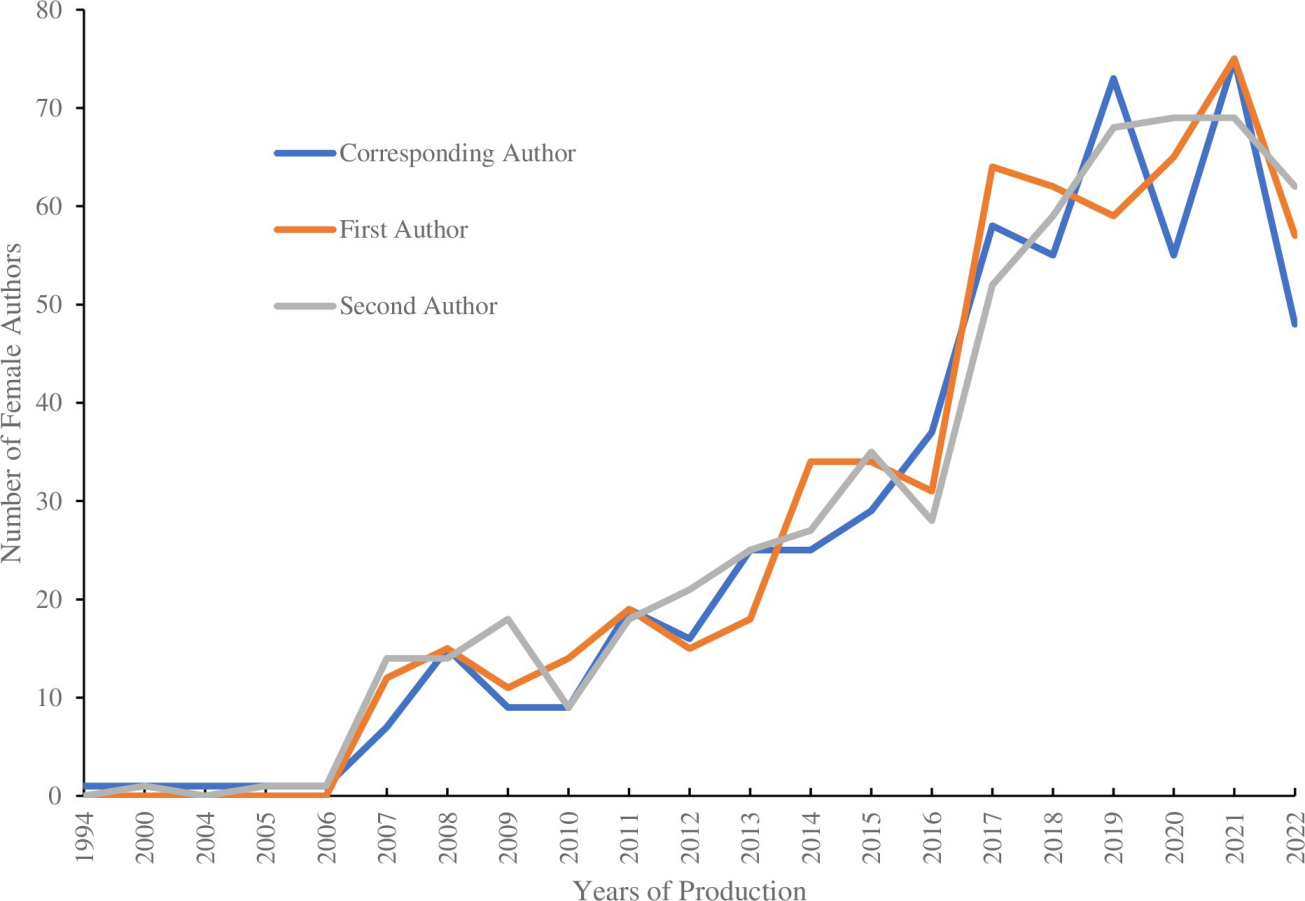
Number of women authors in key positions by year of publication. The double-axis analysis in the figure shows in the vertical axis the evolution in publication years compared to the number of female authors shown in the horizontal axis. The authorship position of the authors in the dataset is indicated by the different colored lines.

The essential characteristics of the temporal dynamics of the absolute and relative position of authors are shown in Fig 4. The graph presents a significant increase in the proportion of female authors appearing in key positions (i.e., first, second, and corresponding) among the total authors. The number of first female authors increased from (1) in 2006 to (75) in 2022, in the case of the second authors increased from (1) to (69) while the number of corresponding female authors changed in the same period from (1) to (55).

In terms of authorship position, significant differences were observed in the corresponding author. Of a total of 1891 publications, 502 were authored by women. The mean number of articles per author also differed according to the gender, with lower production by women than by men. In summary, Table 1 shows a higher proportion of male authors in bioeconomy and rural development studies.

**Table 1:** Authorship production in the bioeconomy and rural development related studies by gender.

Gender	Corresponding Authors (# papers)	Average production (mean of the papers per author) a	Average weight (weight is calculated as an index: author/paper)b
Female	502	1.12	0.85
Male	1389	1.16	0.89

^a^ Average production: the mean value of author’s production in their papers

^b^Average weight: the index of the value of author’s share in their papers.

**Fig 5:**
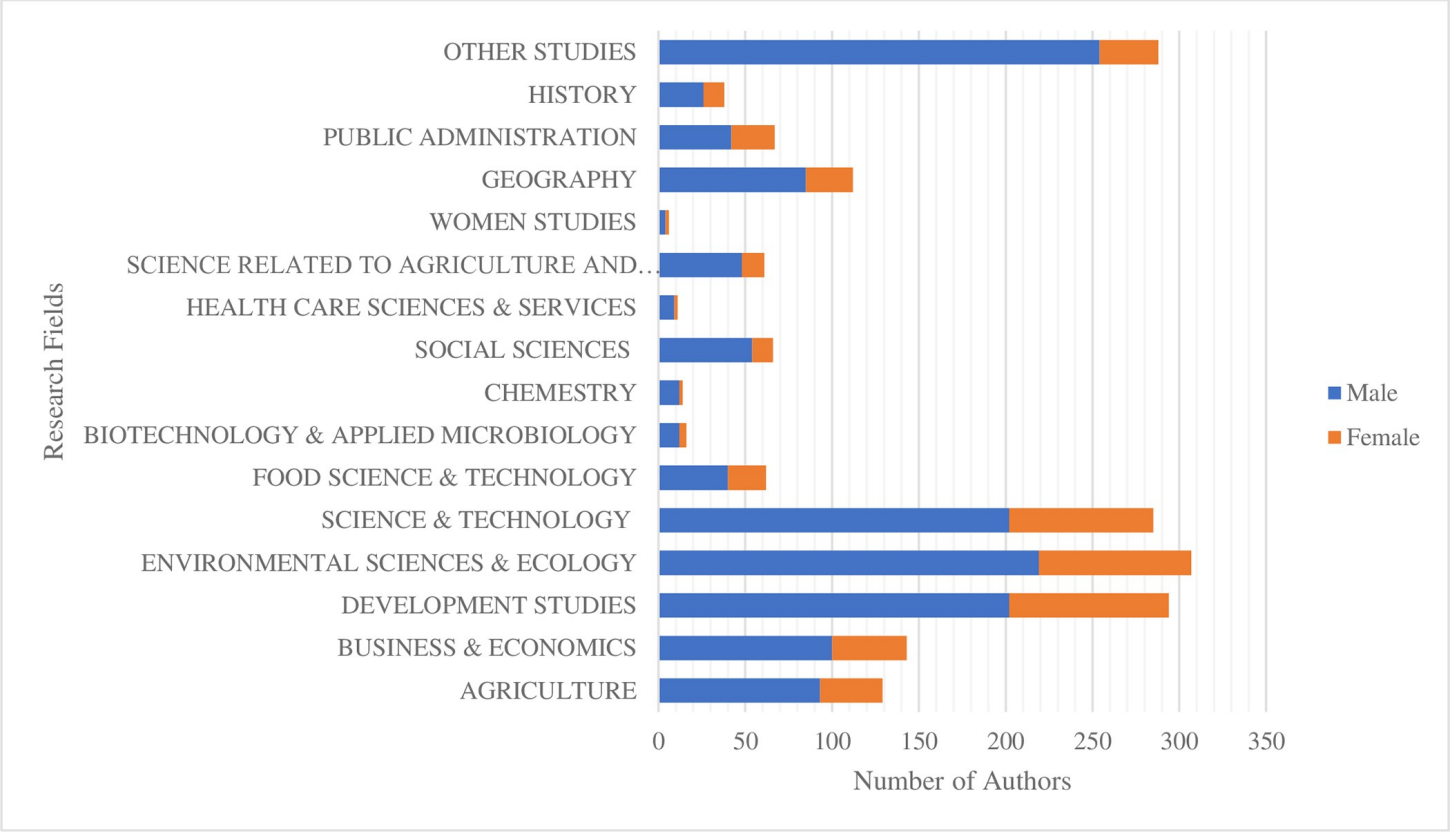
Gender disparities by field of study. By comparing the number of authors in the dataset according to different fields, the graph shows the disparity between male and female authors.

As shown in Fig 5 below, based on our findings, there was a comparison between research work done by males and females in different fields of study mainly related with environmental, development and economics areas. It can be seen that a more significant percentage was carried out by male parties in all the fields of studies with a slight exemption under the women’s studies field.

### Gender distribution based on regions

**Fig 6:**
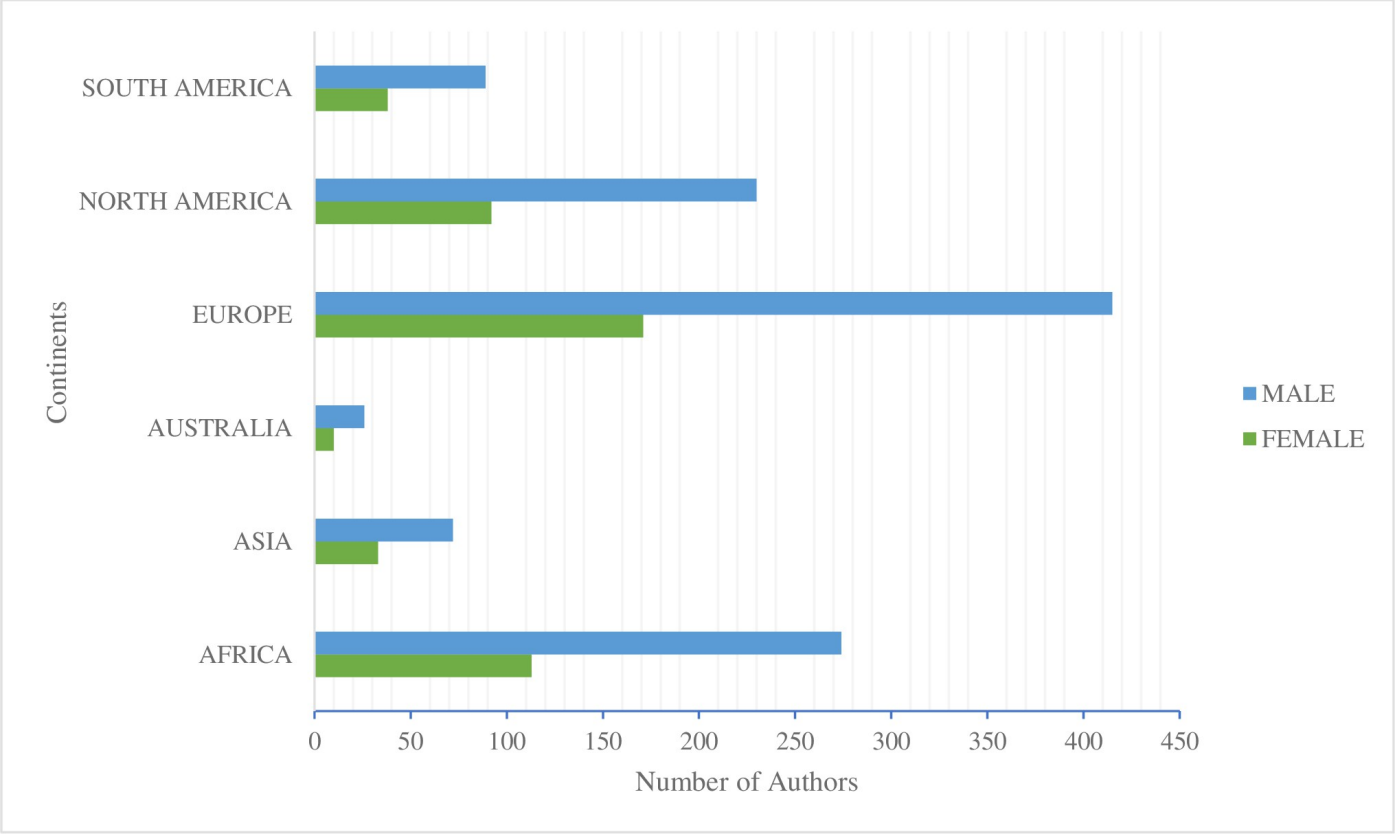
Continental distribution based on gendered division. Based on a double-axis analysis of the number of authors compared to the different continents worldwide. The figure shows the difference between male and female authors.

Gender comparison showed significantly lower involvement of women in research in the world’s different continents than men. Fig 6 revealed that there are more male researchers on all the continents than females in the present study. However, significantly higher numbers are seen in Europe, Africa, and North America, with about (415, 274, and 230), respectively, who have done a study or research in the bioeconomy and rural development field within the collected sample. Nevertheless, if we look at the ratios between the continents, there is a greater degree of parity in regions such as Australia, Asia and South America, where the numerical difference between the genders is smaller than in other continents.

### Annual academic production based on gender

**Fig 7:**
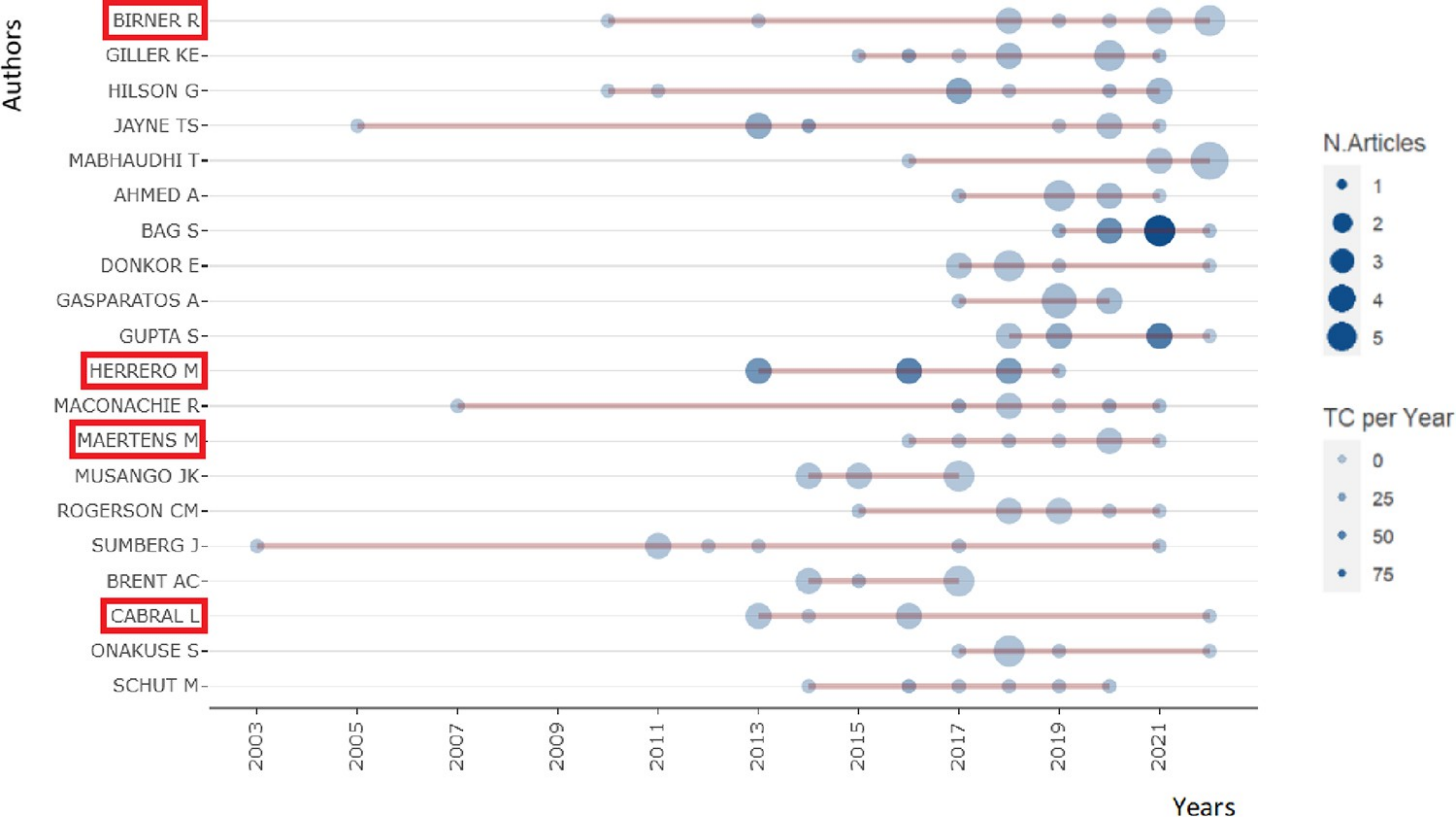
The twenty most productive authors in the field of bioeconomy and rural development in terms of annual scientific production and citations. The graph, created with the biblioshiny software, shows the evolution of the number of publications and the number of citations over the years (vertical axis) of the most productive authors in the dataset (horizontal axis); female authors are highlighted with a red circle.

Fig 7 below, summarizes the 20 most cited authors from the total articles used for this study. Of these authors, just 4 of them are women who are boxed with the red color. This means just about 20% out of the most cited authors are female while the rest are male; it corroborates with our findings above about the skewness and gender biases in the academic’s world. In addition to this, the bubbles explain the number of articles based on the sizes; the more significant the size, the more articles and citations the author has, while the faint red lines tell how long the authors have been producing academic articles, most especially in our concern field of study for this research purpose. The longer the lines are, the older the authors who have been involved in academic writing. Based on the aforementioned statement, our figure represents the gender disparity by finding longer and stronger lines in male authors than in female.

### Distribution of women authorship share

**Fig 8:**
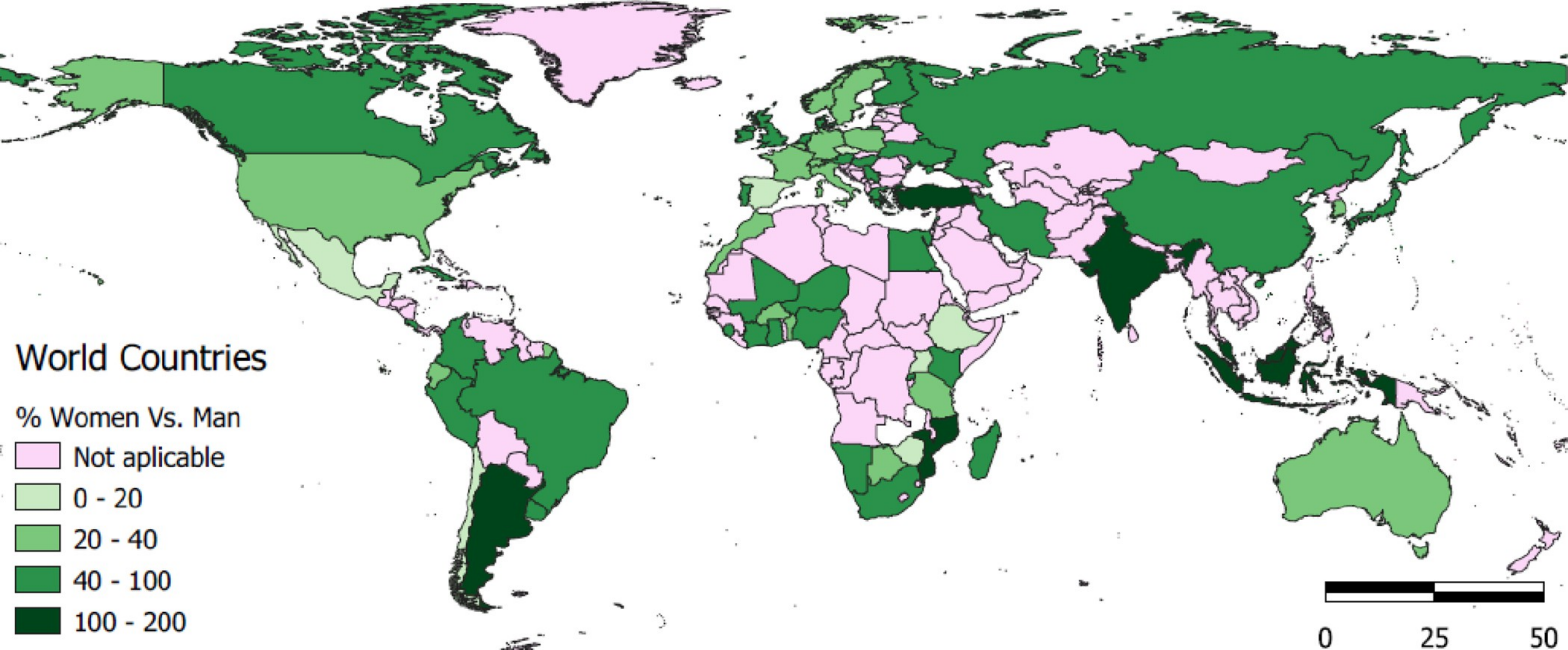
Average share of female corresponding authors in the scientific publications in the data set produced in the different countries (as a percentage of male authors). Made with Natural Earth. Free vector and raster map data @naturalearthdata.com. The figure determines, by means of the grading of the green colors, the percentage of female authors out of the total number of male authors in the data set of the scientific publication. The darker color represents the higher percentage, while the lighter colors represent the lower percentage, the not applicable regions are present in pink.

The world map shown in Fig 8 explains the world distribution of women’s authorship in different continents based on the corresponding authors country affiliation. It is important to note that the countries not shown on the map are those for which no information is available in the database analyzed in this study.

Looking at the African continent, most countries have under 20 percent of women as authors. In comparison, some West African and South African countries like Nigeria and South Africa have more women authorship with about 40 percent and above, which could be because of better exposure of women in research and development As is revealed in Fig 8, majority of these high-income earning countries has a better representation of female in the research field with over 100 percent. Looking at the Latin American continent, there is a better female percentage representation as against Africa, with just a few countries below the 20% range.

### Co-occurrence network analysis

**Fig 9:**
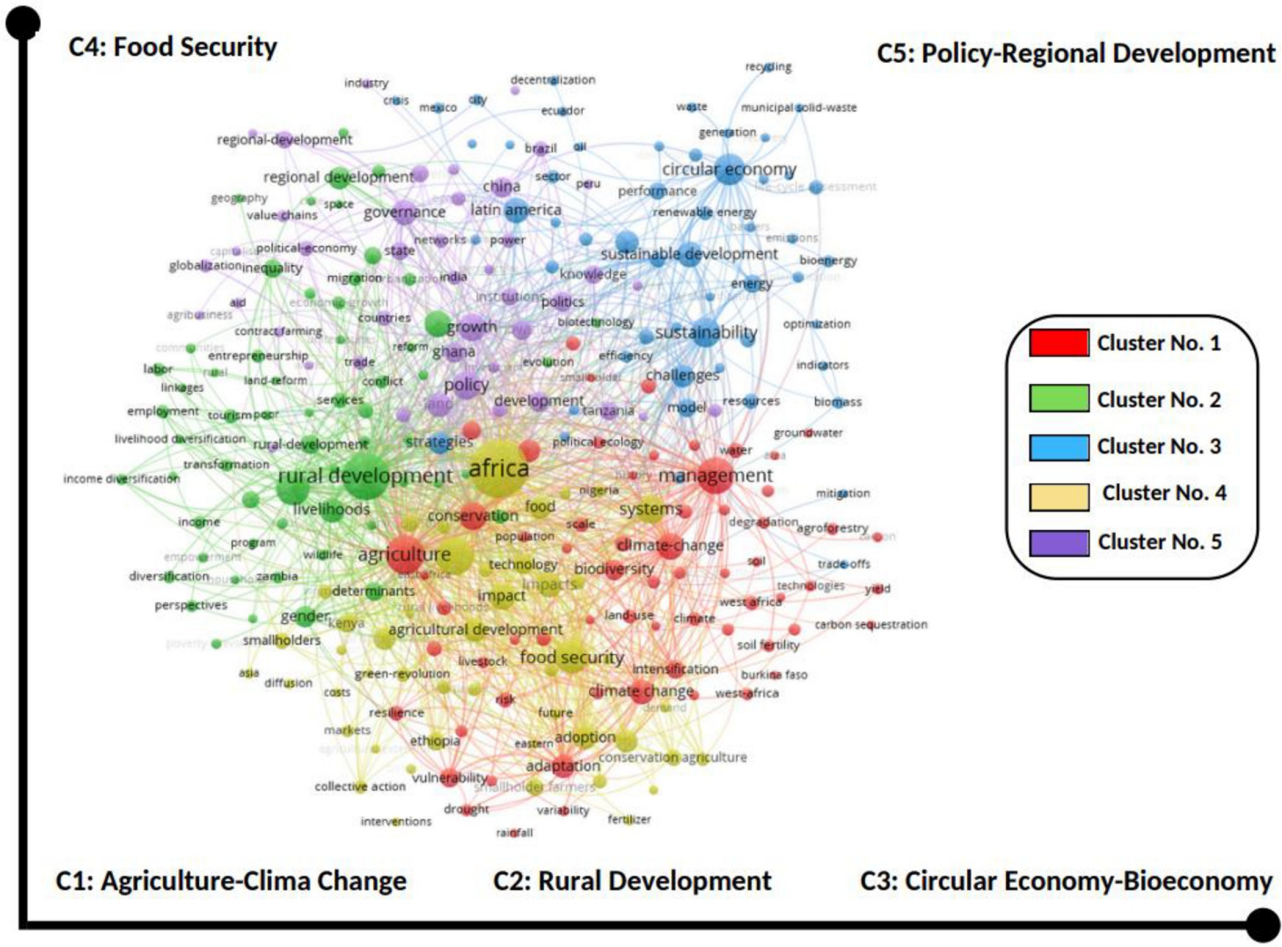
Co-occurrence network of research areas grouped into clusters. The figure shows five clusters based on potential links between all keywords in the dataset. Each cluster groups the pairs of terms related to each other according to a set of criteria defined as co-occurring. (Graphic display with VOSviewer).

Based on the co-occurrence network analysis, the dataset reflects five significant clusters of the study fields (Fig 9). The first one (red color) clusters around 64 items related to agriculture and climate change; these topics play an important role in bioeconomy as agriculture produces most of the biomass feedstock for bioeconomy [54], as well as in the trade-off relationship between natural resource management involving agriculture and its impact of climate change.

The second cluster (green color) contains 61 items and is directly related to rural development and all the inputs and outputs related to this field of study. In the third position, cluster blue, which contains 54 items, focuses on circular economy and bioeconomy, reflecting essential topics such as sustainability, renewable energy, biotechnology, and recycling. These cluster links with the previous ones as traversal. The fourth cluster (yellow color) is directly related to food security and includes essential topics such as population, agricultural development, and conservation.

The final cluster with contains 43 items, is focused on Policy- Regional Development, with has a direct relationship with one of the topics in this research, in here governance, economic growth, state, and globalization are the most Hight light issues that as it was referred previously also have transversal relation with the four-cluster descriptive.

Concerning the thematic structure of the research fields, Table 2 points to important features by gender distribution. The share of articles with at least one-woman author was the highest in the clusters “Rural Development and Policy- Regional Development” (0.30), the second-highest was the cluster related with “Agriculture- Climate change and Bioeconomy” (0.26), and in the last position, the topics related to “Food Security.” In summary, the women’s contribution is almost proportional in most clusters since the difference is not that wide.

**Table 2:** Thematic structure of the research fields and women’s contribution within the clusters.

Cluster names	Size of the cluster (# of items)	Centrality	Density	Share of articles with at least one-woman author
Agriculture—Climate Change	64	0.064	7.016	0.26
Rural Development	61	0.189	4.033	0.30
Circular Economy -Bioeconomy	54	0.110	4.978	0.26
Food Security	47	0.115	5.700	0.21
Policy-Regional Development	43	0.071	4.033	0.30

The degree of centrality and density between the networks (clusters) is also shown in Table 2. Among the clusters analyzed, cluster 2, which is related to rural development, has the highest degree of centrality, followed by food security and bioeconomy. In the case of density, the first and fourth clusters have the highest values.

### Co-authorship network analysis

**Fig 10:**
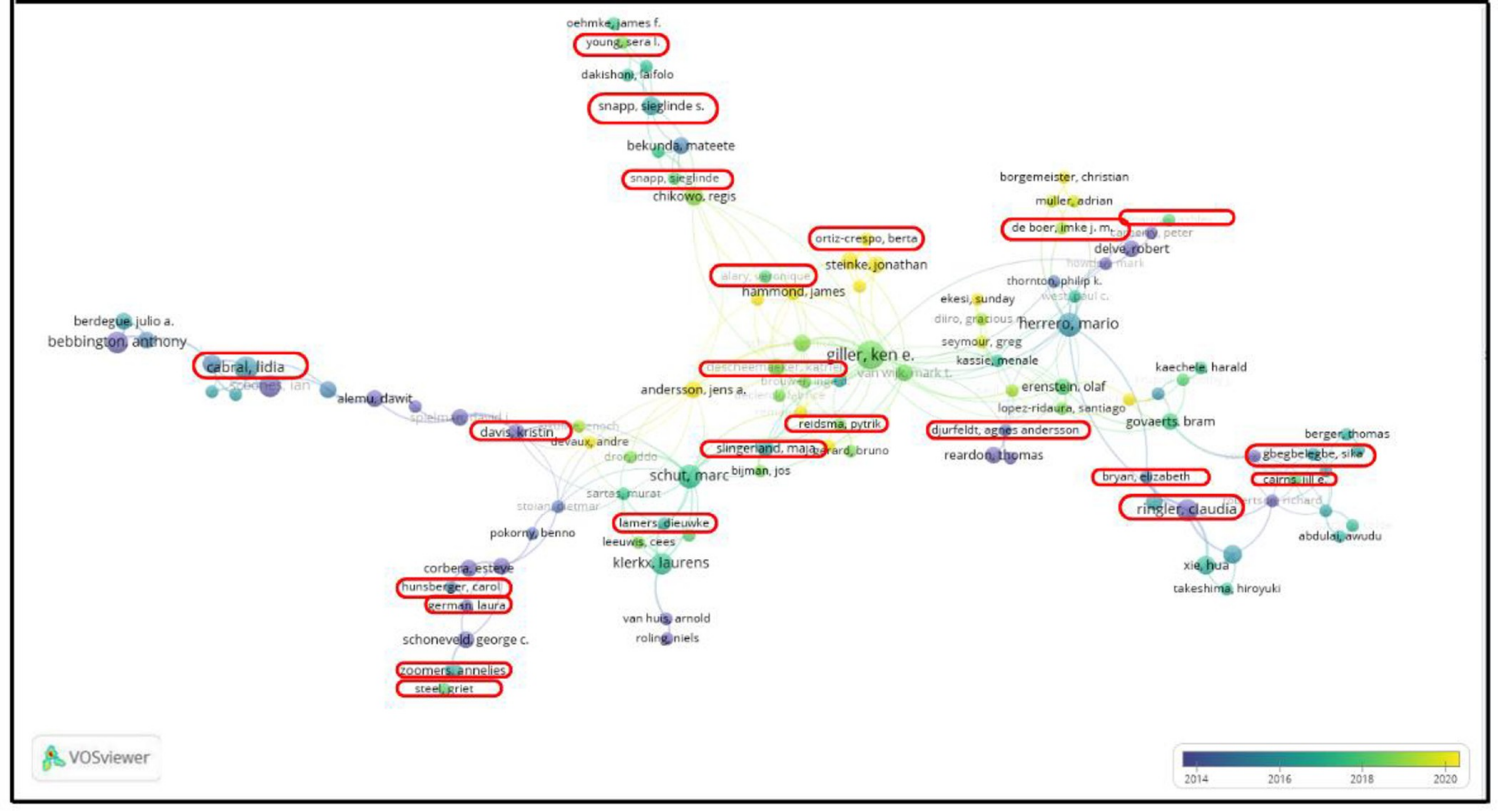
Co-authoring network based on total citations per author. The graph shows the social network in which the authors in the dataset collaborated on one or more publications indirectly linked to each other. The female authors are highlighted in red circles compared to the total number of authors. (Graph displayed with VOSviewer).

To understand the cooperation among the authors, Fig 10 shows the co-authorship analysis, with indicates the relatedness of the items based on the number of co-authored documents.

In this analysis, it is possible to visualize the network of authors based on their co-authored publications; we generate a network of 135 authors. Among all the authors, at least 32 women are noted on the map of research influence and academic impact via shared knowledge and expertise.

To complement the interpretation of the analysis, Table 3 indicates the number of clusters, their size, the number of authors differentiated by gender, the total citations and the centrality measure of the clusters. From 9 of 12 clusters, the male authors are more numerous than females; in one cluster, the relation is equal, and in the last two, no presence of females can be seen. However, in terms of total citations in at least one cluster, the number is higher for females than for male authors (cluster 10).

**Table 3:** Gender co-authorship analysis based on the total citations.

Cluster number	Size of the cluster (# of items)	Closeness	Gender	Number Authors	Average Link Strengths	Total Citations
1	12	0.089	male	7	2.57	568
			female	5	2.4	357
2	12	1	male	11	2.55	1143
			female	1	2.00	139
3	11	1	male	9	2	1095
			female	2	1	143
4	11	1	male	10	1.6	1379
			female	1	2	258
5	10	0.38	male	7	1.86	625
			female	3	1.33	186
6	10	0.2	male	8	2.3	1864
			female	2	2.5	313
7	9	0.26	male	5	2	421
			female	4	1.75	286
8	9	1	male	5	2.2	577
			female	4	2	465
9	8	1	male	6	2.66	374
			female	3	1.5	35
10	6	1	male	3	3	425
			female	3	2.66	1324
11	4	0.39	male	4	1.5	329
			female	0	0	0
12	3	0.5	male	3	2.3	565
			female	0	0	0

In the case of our centrality analysis based on the degree to which a node is close to all other nodes in the network, clusters 2, 3, 5, 8, 9 and 10 are directly connected to all the nodes in the network and also have a higher frequency of male authors than female ones. Clusters 1 and 6 have the lowest values of closeness, where the gender difference is almost half the proportion of female authors compared to male ones.

In order to add more information to the above table, the graph with three fields is analyzed in S1 Fig, in which three elements are presented: list of authors, journals and countries. These elements are plotted with grey links showing how they relate to each other, and the size of each rectangle in each element column indicates the number of articles associated with that element. In the figure, female authors are highlighted with a black square and the numerical difference to male authors is significant. Countries such as South Africa, the UK, the US and Germany dominate scientific research on gender differences in bioeconomy and rural development, followed by Ghana, the Netherlands and Kenya, among others. Importantly, about half of these countries are developed, while others belong to the developing regions. Female authors generally have fewer network links than male authors, although they have more collaborations with countries over the same time period.

## Discussion

The exponential trend in increasing publication in bioeconomy and rural development in both regions that we find in this study may be potentially related to the development of sustainable concepts that have been applied more intensively since the 1970s. In recent decades such themes have evolved to include concepts focused on sustainable rural development, circular economy [55, 56], and since 2014 a global trend of expansion of the instruments of the bioeconomy has allowed to transform the economies of rural areas [28, 57]

Regarding the frequency of publications in the countries of the corresponding authors, according to [58], there is a tremendous disparity in scientific productivity between regions. This finding is corroborated by the study of [59], which points out that female dominance tends to occur in countries with a lower research output, such as South America ones, where there is greater gender parity. Particularly in Latin America, as these countries invest significantly less in science, technology, engineering, and mathematics (STEM) than high-income countries [60–62]. The same logic [63], states that Africa accounts for only 2% of global research output. This is due in part to financial constraints and the high mortality rate of African journals, among other factors.

The result based on the Lotka model and the ANOVA analysis of these data shows that publications in the field of bioeconomy and rural development are closely related in terms of the number of authors, while the distribution of authors and their respective publications shows a strongly skewed pattern, with at least half of the literature produced by the square root of the total number of authors showing no significant difference between men or women. The lack of gender disparity in this specific analysis is also confirmed by [64], whose study shows that there is no difference between male and female productivity when looking at pairs of male and female productivity by subject [64, 65]

These gender differences in the contribution of the women could also be explained by different research outcomes, which also determine the biases faced by female researchers. The study by [60] showed that manuscripts by women are rejected more and published less often than those by men, resulting in fewer scientific opportunities in the future. This is also related to the underrepresentation of women as authors in scientific publications, potentially affecting their representation in academia[66, 67].

Various factors and variables related to socio-cultural parameters at different stages of women’s lives that lead to low participation of women in science may cause this gender imbalance[68]. According to [69], many factors, such as historical, economic, cultural, sociological, psychological, economic and political, foster these inequalities, and therefore gender imbalance is a common phenomenon in developing countries. However, the study by [70], shows that the perception of gender inequalities in science has also been linked to social structures and gendered prejudices and stereotypes, and to revising androcentric knowledge.

The results of this study, with respect to women’s authorship position, are also in line with other studies where it has been observed that women’s perception of being less deserving of recognition in scientific publications has cumulative disadvantages in scientific careers [71–75]. According to [76–78] authorship order is necessary for researchers inside and outside academia for name recognition, prestige and upward mobility in their career. Their study shows a positive change in the proportions of first and last authors over time. However, it also concluded that the most prestigious authorship positions were much more likely to be held by male authors [79] Other findings of the study [80], suggest that women have been historically under-represented in the position of first author and that, in many fields, when it comes to promotion and tenure decisions in academia, authorship order matters. Regarding gender differences in collaboration [72] mentions that "women face greater difficulties in creating informal and professional networks, which may impact on their ability to ask for or receive invitations to collaborate".

In the case of gender equity fields, different approaches suggest that science has at least three types of segregated phases: horizontal, referring to the underrepresentation of women in certain scientific areas; vertical, related to the difficulty of progressing through the levels of the academic career; and, thirdly, the glass ceiling, referring to the limited access to higher positions in the stratification of science [81]. In the first category, segregation causes overrepresentation of women in medical and health fields, social sciences, humanities and administration [82], or in the group of fields called (STEM) where women are a minority [83]. The study of [84] show particularly influential stereotypes in the environment and masculinized culture of scientific fields such as science and informatics. According to [85], most of the disciplines with a gender bias are on their way to parity, but for some of the fields it could be a long time before parity is achieved. The gender bias of Gender Studies is less pronounced, given that women tend to talk more about themselves [86], and may be due to the newness of the discipline. The main exception is economics and law [87], where authors are listed in alphabetical order, which seems to be important for citations, especially if the submission was made to a top economics journal [88]. Our result also confirms that there is a large gap to be filled when it comes to women in research, and highlights the research areas of bio-economy and rural development that are related to this study. In general, gender balance and equality have yet to be achieved in academia, and more needs to be done to create an equal society for researchers in all fields.

Regarding the gender distribution based on regions, our findings are also in line with UNESCO reports, which have shown that in terms of the total number of people employed in R&D, the gender gap is also large globally, revealing that male researchers are more with around 70% of the total compared to the remaining 30% of women [82]. For our study, the region with the highest number of female researchers among the other continents is Europe, the results of UNESCO in 2018 showed that in some European countries the participation of women in R&D is more than 50%. However, in a global context, in the case of gender inequality, according to [89], the correlation between female researchers as a percentage of total researchers and total R&D personnel is negative. This is despite the fact that is know that the higher the total R&D expenditure as a percentage of a country’s GDP, the higher the total R&D personnel per capita [85]. The data from our research highlights the need for a more balanced and inclusive investment in R&D that reduces the gender gap, particularly in the regions of Africa and Latin America, which are the focus of this research. Introducing women to the exciting challenges and opportunities of being a scientific researcher, as suggested by the SGDS on Gender Equality and Social Justice, will promote women’s empowerment [89].

In the case of gender-based annual academic output, for [90] gender differences in overall productivity are smaller for female than for male researchers, especially at the beginning of their careers, with both reaching peak productivity at different points in their lives. However, these differences are offset by the greater productivity leverage that women seem to experience compared to men over time, allowing them to reach productivity parity. In other words, women reach their peak productivity at a later age [91], allowing them to remain productive for longer than men.

If we analyze the distribution of female authorship share in the world in the results of this study, it is possible to ratify that woman in both regions face more challenges in developing their scientific careers than the opposite gender. According to [92] women in Africa have experienced more barriers and difficulties in developing their careers in science, technology and other scientific fields due to lack of funding than women in high-income countries such as Europe and North America. Women researchers in Latin American and Caribbean countries [93], also continue to face numerous challenges, such as discrimination, pay inequality and funding disparities, in pursuing a career in science, discouraging more women from pursuing science. Globally, women are not well represented in research, but there could be an improvement if funding is provided and collaboration of women scientists from different countries and continents is envisaged; this could lead to a fair and balanced participation in the female scientific world.

In the case of the co-occurrence network analysis, it is important to highlight the second cluster (green color) containing "gender" as a vector of inequalities in various dimensions such as in rural employment, which exist and persist due to a number of interrelated social, economic and political factors [91]. The same relevance for third cluster which focus in the sustainability aspects of circular economy and bioeconomy, according to [94, 95] due to climate change and population growth, ethical conflicts are increasing concerning food security by implementing a bioeconomy based on biofuels.

The analysis of the co-authorship network, which represents the cooperation between authors in this study, shows a lower visibility of female authors. According to [96, 97], the loss of talented women in senior academic positions is partly due to the lower number of published articles and the consequent lower visibility of women compared to male scientists. It is important to note that none of the clusters breaks away from the network [98], as all values are greater than zero. This fact has been studied by [99], which indicates that co-authorship patterns are essential in scientific research, as they affect researchers’ individual productivity and, increasingly, funding opportunities. Their findings showed significant differences between male and female researchers in their tendency to publish with a co-authorship [100].

## Conclusion

The objective of this study was to obtain an overview of gender differences in the fields of bioeconomy and rural development based on a rigorous and systematic analysis of the international academic literature. To determine the presence, role, and contribution of women to research output over the last two decades and to obtain an overview of gender trends in the field of bioeconomy and rural development, we applied a combination of different bibliometric approaches and software packages. These provided vital elements to uncover relationships and hidden perspectives of the gender footprint.

### Gender disparities

From our findings, we can suggest that the women in rural development and bioeconomy sciences were under-represented. The number of male authors in the data set was almost three times greater than the number of female authors. The data also show that women’s participation in research has been lower at the regional or continental level. However, regions as South America and Asia have more gender parity than others.

Women’s contributions are considered less relevant than those of male scientists, which translates into a lower number of citations in general and reinforces the invisibility of female researchers. From our dataset, with respect to the twenty most cited researchers in annual scholarly publication, only 20% were women. Apart from that, female authors were more likely to hold other authorship positions than first, second or corresponding.

In terms of the co-authorship network, the most important positions in the network were held by men, showing that women generally find it more difficult to establish a significant network in these male-dominated environments. The presence of women in the co-authorship network showed slight differences, especially in the network indicators. Among the link indicators, the strength of the links indicates almost the same values for women and men, with only in two of the twelve clusters the female link being superior to the male ones.

### Future considerations

We believe it is vital to maintain an open debate on gender-related power dynamics to develop a more equitable distribution of scientific production that considers gender biases and promotes opportunities for female authors to have more visibility within academia. These considerations need to be link with the different factors that may cause these inequalities which are different in each region.

Integrating a feminist approach in bioeconomic and rural development can further enhance knowledge, research, and debate on sustainability by critically assessing and reconstructing these issues, especially in developing regions.

The pursuit of equity in science requires an understanding of the main causes and a holistic approach at the educational, social, economic, cultural, technological, humanistic and other levels with the different actors of civil society, academia and government, especially in regions considered underdeveloped

## Supporting information

S1 Table Author identification gender table-based on gender API analysis. (XLSX)

S1 Fig Three fields plot of journals, authors and countries based on biblioshiny analysis of the database of the study. (TIF)

S1 File Dataset used in the analysis. (XLSX)
