# Supplementary figures and images for: Gender differences in research fields of bioeconomy and rural development-based on sustainable systems in Latin America and Africa regions
Source: PLoS One. 2024 Aug 22;19(8):e0308713. doi: 10.1371/journal.pone.0308713 (PMC11340968; doi:10.1371/journal.pone.0308713)

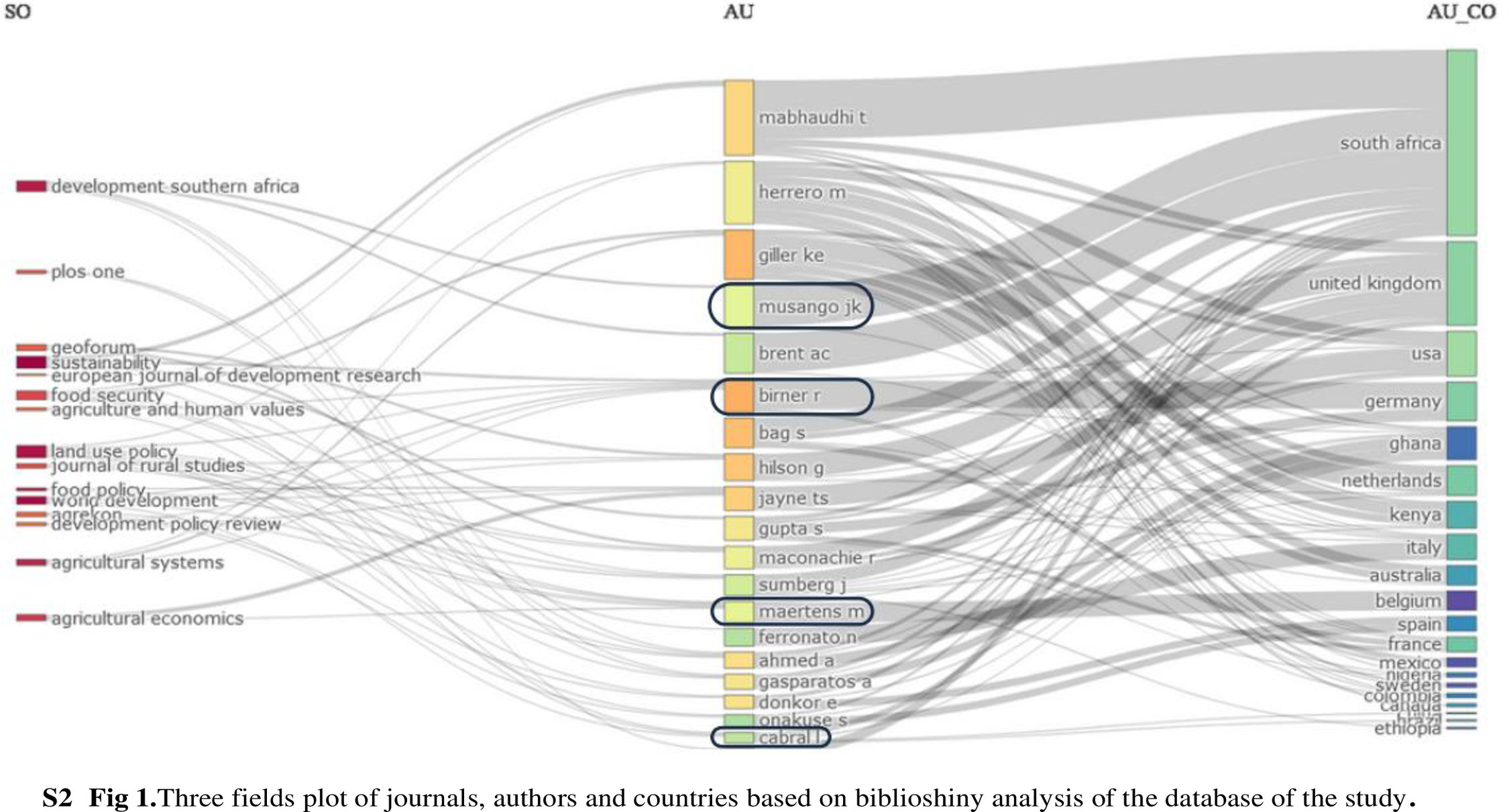

Supplement: S1 Fig — (TIF) [file pone.0308713.s002.tif]
